# A Video-Based Behavioral Intervention Associated with Improved HPV Knowledge and Intention to Vaccinate

**DOI:** 10.3390/vaccines10040562

**Published:** 2022-04-05

**Authors:** Sarah Marshall, Anne C. Moore, Aoife Fleming, Laura J. Sahm

**Affiliations:** 1 Pharmaceutical Care Research Group, School of Pharmacy, University College Cork, T12 K8AF Cork, Ireland; 104310659@umail.ucc.ie (S.M.); a.fleming@ucc.ie (A.F.); 2 School of Biochemistry and Cell Biology, University College Cork, T12 K8AF Cork, Ireland; anne.moore@ucc.ie; 3 Department of Pharmacy, Mercy University Hospital, T12 WE28 Cork, Ireland

**Keywords:** HPV, feasibility study, vaccine knowledge, behavioral intervention, intention to vaccinate

## Abstract

The aim of this study was to design, develop, and evaluate the feasibility of a theory- and evidence-based intervention to improve human papillomavirus (HPV) and HPV vaccine knowledge and intention to vaccinate, among parent–daughter dyads. A theory- and evidence-based online behavioral intervention, “*Is the HPV vaccine for me?*”, was developed to improve HPV and HPV vaccine knowledge and intention to vaccinate. Knowledge, intention to vaccinate, and feasibility of the intervention were evaluated in a prospective, randomized, controlled feasibility trial. A total of 49 parent–daughter dyads completed the baseline knowledge assessment (*n* = 24 control, *n* = 25 intervention), and 35 dyads completed the knowledge assessment at week 2 (*n* = 17 control, *n* = 18 intervention). The intervention resulted in a statistically significant increase in HPV and HPV vaccine knowledge and intention to vaccinate. All intervention participants found the video interesting, while 96% found it useful. This intervention was found to be useful, effective, safe, and acceptable in this feasibility study.

## 1. Introduction

HPV is responsible for approximately 4.5% of global cancer disease burden, with cervical cancer the most common cancer caused by HPV infection [1]. Three vaccines are licensed and marketed for use to prevent HPV infections and their sequelae: Cervarix^®^, Gardasil^®^, and Gardasil 9^®^. In the adolescent population, these vaccines are intended to be administered before the onset of sexual activity [1]. The safety of these vaccines is well established [2]. However, unsubstantiated claims linking the administration of these vaccines to the development of a plethora of adverse effects, as per reference [1], has led to a significant reduction in vaccine uptake worldwide [3]. There is, therefore, a need to develop interventions to support positive vaccine decision making [4]. Several interventions have been designed to address HPV vaccine hesitancy, frequently targeting parents [5]; evaluation is often based on the impact of the intervention on parents’ intention to vaccinate, with several studies reporting a statistically significant impact on intentions [6,7]. However, it has been recommended that adolescents be included in healthcare decision making [8]. Therefore, in this study, the target population of our behavioral intervention was parent–daughter dyads.

Interventions have been shown to be more effective if they are based on principles drawn from evidence and theories of behavior change [9]. We generated the evidence for the current intervention in a comprehensive systematic review as per reference [10] and through a series of qualitative research studies on both female adolescents [11] and parents (Table 1). Our qualitative studies were guided by behavior change theory, including the Theoretical Domains Framework (TDF), reference [12] the COM-B model [13], the Behaviour Change Wheel (BCW) [13], and the Behaviour Change Technique Taxonomy version 1 (BCTTv1) [14]. This methodology has been successfully applied to develop several health behaviour interventions including sexual health/contraception [15], smoking [16,17], diet, and exercise [18,19,20,21,22,23,24]. The TDF guided the development of a focus group and interview topic guides and was used as a coding framework in data analysis [13]. Ten of the 14 TDF domains were selected as the most relevant [12,25].

The 10 TDF domains were linked to several components of the COM-B model: psychological capability, physical and social opportunity, and reflective and automatic motivation. The BCW was then used to identify five relevant intervention functions: education, persuasion, environmental restructuring, modelling, and enablement, as per reference [26] which were linked to 15 appropriate BCTs [14] (Table 2).

In this study, we examined the feasibility of our intervention and the feasibility of performing an RCT in a relevant population, as a recommended implementation strategy [27,28]. Findings will inform the decision to conduct a future RCT and will provide proof-of-concept evidence. As behaviours relating to information were frequently identified in our previous qualitative investigations (Table 2), the aim of this study was to design, develop, and evaluate the feasibility of a theory- and evidence-based intervention to improve HPV and HPV vaccine knowledge and intention to vaccinate among parent–daughter dyads. The key objectives were as follows: (1) to assess the feasibility of the intervention by assessing participation rates and increase in knowledge and (2) to generate proof-of-concept evidence of the effect of the intervention on intention to vaccinate and any possible unintended consequences of the intervention

## 2. Materials and Methods

Ethical approval was obtained from the Social Research Ethics Committee, University College Cork (Log 2019-26). An online behavioural intervention, titled “*Is the HPV vaccine for me?*”, was developed. The 6-min video was created using VideoScribe 3.3.1-1 software by Sparkol^®^, in consultation with a Technology-Enabled Learning Co-ordinator. A narrative approach was applied, mapping the adolescent HPV vaccine decision journey (BCT: demonstration of the behaviour; social comparison). It was narrated by the primary researcher, and definitions and numerical information were complemented by graphical illustration. The information provided was evidence based and theoretically informed, bridging the knowledge gaps identified through previous research [11]. It addressed the objectives outlined in Table 3, according to the identified BCTs. The video finished with a reminder that most girls in Ireland accept the HPV vaccine (BCT: information about social and environmental consequences, social comparison, information about others’ approval).

A prospective, randomized, controlled feasibility trial (RCT), containing an intervention group that had access to the video and a control group that did not have access to the video, was conducted to evaluate the intervention in Cork, Ireland. Eligible participants were parent–adolescent dyads including a female adolescent, pre-HPV vaccination, in her final year of primary school (ISCED level 1), as per reference [29] typically aged 11–12 years. Recruitment took place over a 6-week enrolment period from April to May 2019. A list of primary schools was compiled and stratified according to DEIS (Delivering Equality of Opportunity in Schools) status [30]. The DEIS program supports children who are at greatest risk of educational disadvantage [30]. Using a purposive sampling strategy, school principals were contacted via email and/or telephone and provided with details of the trial. An invitation to all schools in Cork County was proposed to ensure a representative sample of DEIS and non-DEIS schools and to account for low recruitment and loss. Schools interested in participating were then randomized by simple randomization. The principals were asked to share study information, via email, with eligible participants. This email detailed trial information, expectations of participation, and instructions on accessing the trial material. Google Forms was used as the data collection platform, recording consent to participate and baseline characteristics of the parent: gender, age range, highest education level achieved, number of children (under 18 years), and vaccination status of children.

**Figure 1:**
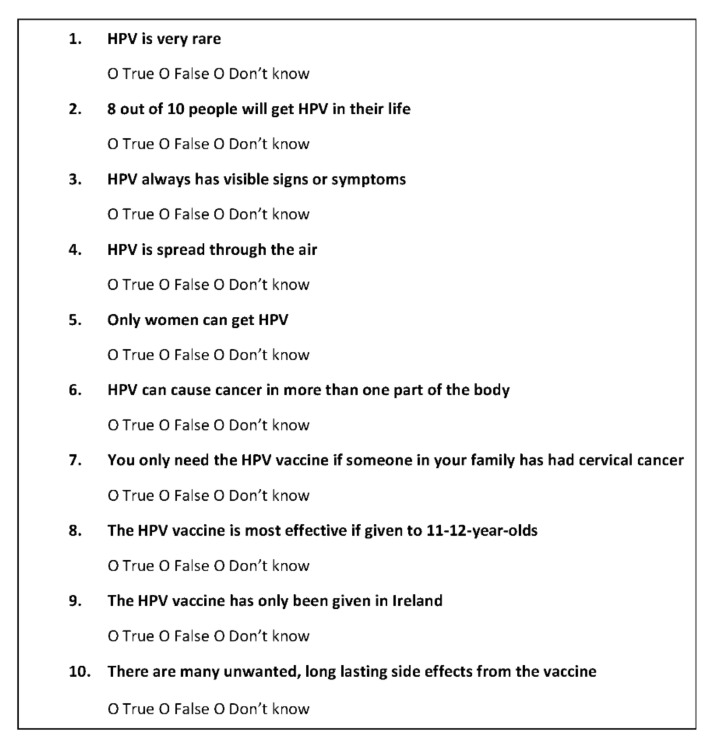
HPV and HPV vaccine knowledge questionnaire.

A 10-item questionnaire was developed to evaluate participants’ baseline HPV and HPV vaccine knowledge and intention to vaccinate (Figure 1). The items assessed knowledge using a “True, False, Don’t know” format. These were identified as knowledge gaps during a previous literature review and qualitative research [10,11]. The questionnaire was developed, edited, and assessed for face and content validity but did not undergo external validation. A knowledge score was based upon correct responses to items, with 1 point being awarded for each correct response obtained (range 0–10). No points were rewarded for “Don’t know” responses. Participants were also asked about their intention to accept the HPV vaccine (“Yes, No, Don’t know”). This question was not scored. At the outset of the study (W0), all participants undertook baseline knowledge assessment. Those in the intervention group were immediately invited to view the video. Two weeks later (W2), all participants repeated the knowledge assessment. Those in the intervention group were asked whether the video had increased the likelihood of accepting the HPV vaccine (“Yes, No, Don’t know”). After completing the knowledge assessment at W2, those in the control group were provided the opportunity to view the video. At W2, participants in the intervention group were asked the following questions: “Did you find this video interesting?” and “Did you find this video useful?” (“Yes, No, Don’t know”). In addition, feedback from participants was obtained through the provision of a free-text box.

As this was a feasibility trial, a formal sample size calculation was not necessary. Our aim was to recruit a sample to give us reasonable confidence in our decision to progress to a full RCT. Our sample size justification was based on the CONSORT 2010 guideline that the rationale for the sample can include *assessment of practicalities and estimation rates or rationale based on the percentage of number required for the future definitive RCT* [31,32].

Data were analyzed using IBM’s SPSS or GraphPad Prism. Continuous variables were described by medians and IQRs (non-parametric data). Categorical variables were described by counts and percentages. Associations between categorical variables were investigated using Fisher’s exact tests (two sided). A one-way ANOVA was used to investigate differences between groups for continuous variables. The *p* values of <0.05 were considered statistically significant.

## 3. Results

A total of 313 schools were invited to participate (*n* = 37 DEIS, *n* = 276 non-DEIS). Eleven schools agreed to participate (*n* = 4 DEIS, *n* = 7 non-DEIS) and were randomized (*n* = 5 control, *n* = 6 intervention). According to information provided by schools, 326 parent–daughter dyads were eligible to participate. A total of 49 dyads completed the baseline knowledge assessment at week 0 (W0) (*n* = 24 control, *n* = 25 intervention), resulting in a response rate of 15.03%, and 35 dyads completed the knowledge assessment at week 2 (W2) (*n* = 17 control, *n* = 18 intervention), for a response rate of 10.74%. All participants were female and declared that, to the best of their knowledge, all children in their care were fully vaccinated. Using Fisher’s exact test, there were no statistically significant differences between the control and intervention groups based on DEIS status (*p* = 0.773), age of parent (*p* = 0.089), education level of parent (*p* = 0.49), number of children (*p* = 0.321), or intention to accept the HPV vaccine (*p* = 0.778).

At W0, the median (IQR) baseline knowledge score was 5 (4, 6). There was no statistically significant difference in the baseline knowledge assessment scores between the control and intervention groups (*p* = 0.870). Just over half (51%) of the participants indicated that they intended to accept the HPV vaccine, while the remaining 49% remained undecided.

**Figure 2:**
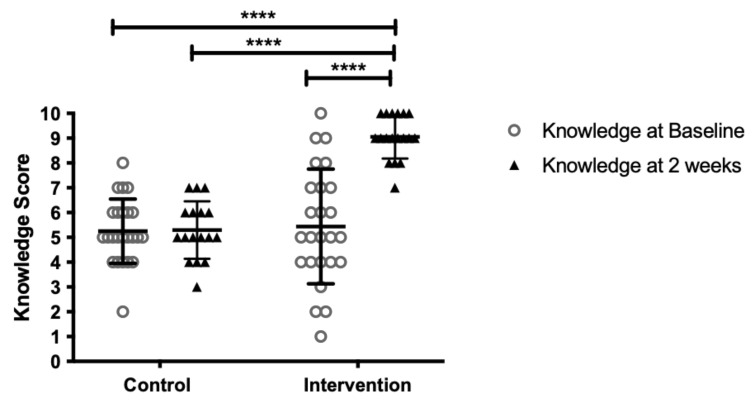
Knowledge Assessment Scores at baseline and at 2 weeks in the control (*n* = 25 at baseline, *n* = 17 at week 4) and intervention (*n* = 25 at baseline, *n* = 18 at week 4) groups. Bars represent the mean and standard deviation; **** *p* < 0.0001 as assessed by one-way ANOVA.

Two weeks later, there was a statistically significant difference in the knowledge assessment scores between the control and intervention groups (Figure 2). When asked whether this video had increased the likelihood of accepting the HPV vaccine, 88% indicated that it had, 4% indicated that it had not, and 8% were unsure. All intervention participants found the video interesting, while 96% found it useful.

## 4. Discussion

The purpose of this study was to design, develop, and evaluate the feasibility of a theory- and evidence-based intervention to improve knowledge about HPV and HPV vaccines and intention to vaccinate among parent–daughter dyads. It was intended that targeting these dyads would promote open dialogue between parent–daughter pairs, leading to a scenario where the adolescent was involved and participated in the vaccine decision. While several interventions have been designed to address HPV vaccine hesitancy [6,7,33,34,35], there are no published examples, using the BCW to develop a *de novo* online intervention, targeted at parent–daughter dyads. The chosen mode of delivery was an online video. Digital media has several advantages: videos can be entertaining, their medium is familiar, and they may be designed as a “takeaway tool” that permits more independent application at the viewer’s own pace [36]. We found that this educational intervention significantly increased knowledge for participants who viewed the video in this cohort of 49 parent–daughter dyads. Secondly, we found that this video increased the likelihood of accepting the HPV vaccine for most participants. However, this study was not designed to examine a subsequent vaccination rate. This study provides an initial “proof of concept” that an educational intervention designed from a solid foundation of behavior change theory can positively influence HPV vaccine decision making. Further studies are required to understand the association between this increase in knowledge and HPV vaccine uptake.

The intervention was designed and evaluated using the APEASE criteria: a set of criteria used to make context-based decisions on intervention content and delivery consisting of affordability, practicability, effectiveness and cost effectiveness, acceptability, side-effects/safety, and equity considerations [13]. While affordability is difficult to quantify in this case, this video-based intervention was created in consultation with a Technology-Enabled Learning Co-ordinator, with minimal financial input. This video was hosted free of charge on a YouTube^®^ platform (unlisted). This intervention could be adopted in its current form with no further financial investment. It is practicable in its mode of delivery: the video is hosted online, and the internet is used as a platform to disseminate the content to the target population. According to data collected in 2019 by Ireland’s Central Statistics Office (CSO), an estimated 91% of Irish households had access to the internet at home, with 57% of individuals seeking health-related information online [37]. More people are accessing internet-based content by following links on social media than through direct searches [38]. Social media statistics from June 2018 indicated that up to 66% of Irish individuals (over 15 years) were using social networking sites (e.g., Facebook^®^, Instagram^®^, LinkedIn^®^, Twitter^®^) [39]. It has been demonstrated that information shared via social media results in a greater knowledge transfer than when shared via pamphlets [40]. In addition, it has been postulated that social media has a direct public health relevance because social networks could have an important positive influence on health behaviours and outcomes [41,42]. Therefore, social media platforms have the potential to effectively increase knowledge and facilitate behaviour change. The intervention described in this study is readily amenable to dissemination on social media platforms.

In this small feasibility study, we defined the baseline of knowledge on HPV vaccines across these participants and we determined that the video successfully and significantly increased knowledge for these participants. Secondly, at the outset, 49% of participants were undecided about their vaccine decision. Furthermore, on completion of the study, 88% of participants indicated that the video had increased the likelihood of accepting the vaccine. While this was a positive finding, it must be acknowledged that intention alone does not necessarily predict future vaccine uptake, as per reference [43] a disparity known as the intention–behaviour gap [44]. A variety of strategies have been suggested to bridge this gap: keeping these favourable immunization intentions in mind through reminders, prompts, and cues and reducing barriers through logistics and heuristics [45]. While it would have been desirable to evaluate the impact of this intervention on actual vaccine uptake, this was not an objective of the study. Due to the affordability, practicability, and effectiveness of the intervention, it was determined to be cost effective.

There were no statistically significant differences between groups according to DEIS status, age range, education, number of children, child vaccination status, and intention to accept the HPV vaccine. Although all parent participants were female, such a gender imbalance is not unusual. Research has demonstrated that the female care-giver is more likely to participate in clinical research as shown in reference [46] and is often the primary healthcare decision maker for the family [47].

Acceptability has become a key consideration in the design, evaluation, and implementation of healthcare interventions and is a necessary condition for effectiveness [48]. The acceptability of this intervention was evaluated: all respondents found the video interesting, while 96% found it useful. However, only 3.5% of the invited schools consented to participate. While an effort was made to understand the reasons underpinning their lack of participation, most contacted schools were non-responsive. Of those eligible to complete the knowledge assessment at W0, only 15% did so, and this was further reduced to 10.7% at W2. This decline in the response rate between phases is frequently observed [49]. However, a higher response rate was expected due to the personal relevance of the research topic [50]. This intervention was delivered in May, i.e., 4 months before the vaccine would be offered to the participants, to permit a timely provision of vaccine information [51]. It is possible that the time lag between information delivery and actual vaccination date was too long, participants were not yet prepared to consider their vaccine decision, and, thus, were not personally invested in the intervention content. Therefore, the resultant sample size was small, and it is possible that the findings may not be readily generalizable to the wider population. However, the participants were sampled across a geographical area and across a range of socio-demographic environments. An intervention may be effective and practicable but have unwanted side effects or unintended consequences [13]. Research has shown that information provision regarding vaccine safety and efficacy can cause unpredictable effects on vaccination uptake and may even increase such concerns [52,53]. A free-text box was provided in this study and no participants reported any such concerns. However, the potential for such an occurrence if the intervention were to be scaled up should be considered.

The use of video as a mode of delivery facilitates equity as it provides standardized content across learners and has been shown to be effective among viewers of lower literacy levels [54]. However, the impact of the ‘digital divide’ on the implementation of an online intervention must be considered. A proportion of the Irish population (9%) does not have internet access at home with 42% of these reporting that a lack of skills hampered their internet access [38]. Parents (and adolescents) currently receive vaccine information in written format (i.e., pamphlets and patient information leaflets (PIL)). Additionally, the website of the National Immunisation Office (NIO) is signposted, providing further information in a variety of formats. The intervention described here is not intended to replace such material, but rather to support and complement it, providing information and promoting behaviour change. This feasibility study to investigate how this intervention could be rigorously tested and implemented informs the development of future studies with respect to recruitment rates and initial information on inter-group variability and effect size in a future interventional trial. This study did not aim to assess all barriers and strengths to implementation of the suggested intervention. While this was a small feasibility study, it was evidence-based and used the rigor of behavior change theory relating to both adolescents and parents.

## 5. Conclusions

A video-based online behavioural intervention was associated with improved HPV (and HPV vaccine) knowledge and intention to vaccinate among parent–daughter dyads. The intervention was found to be useful, practicable, effective, acceptable, and safe in this feasibility study. It is important to acknowledge that vaccination is highly context specific [55]. Therefore, the impact of this intervention will need to be evaluated in alternative contexts. In addition, since September 2019, male adolescents were included in the HPV vaccination program (1). An assessment of the impact of this intervention in parent–son dyads is required, making alterations as required and supplementing with further qualitative research, if indicated. Should this intervention demonstrate efficacy across multiple contexts, a national dissemination of “*Is the HPV vaccine for me?*” could positively impact decision making with respect to HPV vaccination in Ireland.

## Figures and Tables

**Table 1:** Summary of Theoretical Domains Framework (TDF) domains identified in parents and adolescents through previous qualitative research.

TDF Domain	Parents	Adolescents
Knowledge	✓	✓
Memory, attention, and decision processes	✓	
Social role and identity	✓	
Beliefs about capabilities	✓	✓
Optimism	✓	✓
Beliefs about consequences	✓	✓
Goals	✓	
Emotion	✓	✓
Environmental context and resources	✓	✓
Social influences	✓	✓

**Table 2:** Summary of Behaviour Change Techniques (BCT) identified in parents and adolescents through previous qualitative research.

BCT	Parents	Adolescents
Information about consequences	✓	✓
Salience of consequences	✓	✓
Information about social and environmental consequences	✓	✓
Anticipated regret	✓	✓
Information about emotional consequences	✓	✓
Demonstration of the behavior	✓	✓
Social comparison	✓	
Information about others’ approval	✓	
Prompts/cues	✓	
Credible source	✓	✓
Pros and cons	✓	✓
Comparative imagining of future outcomes	✓	
Restructuring the physical environment	✓	✓
Focus on past success	✓	✓
Identification of self as role model		✓

**Table 3:** Intervention objectives and associated Behaviour Change Techniques (BCTs).

Intervention Objectives	BCTs
Understand how HPV is transmitted	Information about health consequences
Know how common HPV infections are	Information about health consequences; Salience of consequences
Know that HPV infects both men and women	Information about health consequences
Understand the consequences of long-term HPV infection(s)	Information about health consequences; Salience of consequences; Anticipated regret; Information about emotional consequences; Comparative imaging of future outcomes; Pros and cons
Understand why the vaccine is administered at the recommended age	Information about health consequences
Appreciate the safety and efficacy of the vaccine	Credible source; Focus on past success
Know the vaccine side effects	Credible source; Pros and cons

## Data Availability

Not applicable.
